# Increased Physical Fitness Is Associated with Higher Executive Functioning in People with Dementia

**DOI:** 10.3389/fpubh.2017.00346

**Published:** 2017-12-21

**Authors:** Alice Hollamby, Eddy J. Davelaar, Dorina Cadar

**Affiliations:** ^1^Department of Behavioural Science and Health, University College London, London, United Kingdom; ^2^Department of Psychological Sciences, Birkbeck College, University of London, London, United Kingdom

**Keywords:** dementia, aging, physical activity, physical fitness, cognition

## Abstract

Physical fitness (PF) has been associated with improved cognition in older age, but less is known about its effects on different cognitive domains in individuals diagnosed with dementia. We explored the associations between PF and cognitive performance in 40 healthy elderly and 30 individuals with dementia. Participants completed a battery of standardized cognitive tests (Mini-Mental State Exam, Verbal Fluency, Prospective and Retrospective Memory Questionnaire, Clock Drawing, and California Verbal Learning Test) and were classified into high versus low levels of PF based on their score on the Physical Fitness Questionnaire. Analyses took into account age, gender, education, occupation, head injury, Internet use, brain training, and past levels of exercise and revealed overall benefits of PF, in particular for the people with dementia. Discriminant analysis showed high accuracy of reclassification, with most errors being due to the misclassification of dementia cases as healthy when they had high PF. The first discriminant function accounted for 83% of the variance. Using individual estimates of this function, which reflected global cognitive performance, confirmed the beneficial role of PF in dementia, even when taking into account age, past level of exercise, and the number of years since the dementia diagnosis. Finally, univariate analyses confirmed the differential sensitivity of the cognitive tests, with MMSE and clock drawing showing reliable interaction effects. This work shows that PF is associated with a reduced level of cognitive deterioration expected with dementia, especially in executive functioning and provides empirical support for the cognitive benefits of interventions promoting PF for individuals with dementia.

## Highlight

Physical fitness is associated with higher global cognition in individuals with dementia, independent of past levels of exercise and illness duration.

## Introduction

Dementia is one of the leading causes of disability in later life, ahead of cancer, cardiovascular disease, and stroke (1) and is characterized by a decline in many aspects of cognitive ability. Despite promising new research (2), there is currently no pharmacological cure for dementia (3). It is, therefore, important to help identify behavioral and environmental modifiable risk factors that could reduce the progression of the disease. One such risk factor is physical activity (PA), which is defined as any bodily movement produced by skeletal muscles that result in energy expenditure. PA differs from physical fitness (PF), which represents a set of attributes that people have or achieve that relates to the ability to perform PA.

Current recommendations from the World Health Organization (4) suggest that throughout the week, older adults (age 65+) should perform either at least 150 min of moderate-intensity aerobic PA or 75 min of vigorous-intensity aerobic PA. The National Institute for Health and Care Excellence recommends that adults increase their PA levels to reduce the risk of dementia (5), but does not recommend guidelines for the amount of exercise recommended for people with dementia.

Previous research has suggested increased levels of PA as an intervention for maintaining cognitive performance in older adults (6, 7); for reviews, see Ref. (8, 9). Results of a meta-analysis of 29 randomized control trials (RCTs) found that sustained PA was associated with improved scores in memory, attention, and processing speed in both healthy adults and those with mild cognitive impairment (MCI) (10). Similarly, a recent cross-sectional study of 7,000 older adults found that higher levels of objectively measured PA were associated with better performance in memory and executive function and lower prevalence of cognitive impairment (11). However, not all research has supported this association. For example, Young et al. (12) reviewed 12 RCTs of 750 participants and found that aerobic PA to have no cognitive benefit in cognitively healthy older adults.

The effect of PA has also been investigated in people with dementia, with a substantial body of research reporting a positive association between increased levels of PA and improved cognition [for reviews, see Ref. (13–16)]. A RCT of 185 older adults with dementia found a significant effect of PF on dual-task performance (e.g., 10-m walking while counting backwards from 50 or naming the months backwards) and other tests of cognition [e.g., Mini-Mental State Examination (MMSE), Symbol Digit Modalities Test, the Stroop Colour and Word Test, and Lexical Verbal Fluency Test] (17). However, not all research has supported this positive effect. Forbes et al. (18) found no significant impact of exercise programs on improving cognitive function in a review of 17 RCTs studying 1,067 older adults with dementia, but did notice improvements in activities of daily living. Beyond improving outcomes for people with dementia, a systematic review of 26 studies reported a 14% reduction in dementia risk for those with high PA levels (RR 0.86, 95% CI 0.76–0.97) compared with those inactive (19), see also Ref. (20–22).

The overall findings suggest an advantage of PA on cognitive ability even in individuals with dementia, but some of the present inconsistencies indicate a highly complex interaction between the level of PF, the type of PA, and the neuropsychological domains measured in various studies. In particular, studies found selective improvements in executive function rather than general cognitive benefits due to PA (23–25). Therefore, it is recommended to use a battery of neuropsychological tests rather than a single measure of cognition (14, 26). For example, in a study that used multiple cognitive tests in oldest-old women (self-reported), PA was associated with better scores in the modified MMSE, category fluency, and Trails B, but not for digit span backwards, phonemic fluency, and delayed recall at 5 years follow-up (27). In another study, Matthews and Williams (28) found that Tai Chi was effective at improving performance in the Trails B and clock drawing tests, but not in the digit-symbol substitution test. Interactions between age and PA were also found for measures of immediate memory and visuospatial ability, and a trend for verbal ability (29).

Although most of the aforementioned work has focused on PA, there is less research addressing the role of PF in cognitive performance in dementia. A review of 16 intervention studies revealed improvements in physical functioning irrespective of the stage of dementia (30). For example, Santana-Sosa et al. (31) demonstrated improved PF after a 12-week exercise intervention period, but they did not assess cognitive function. However, Huh and colleagues (32) observed a cross-sectional association between executive functioning and PF, regardless of PA level. As of yet, there are no studies looking at both PA and PF on cognitive performance in dementia. Given the importance of ascertaining the potential differential contributions of PA and PF, more research is necessary to evaluate these specific associations with cognitive functioning, particularly among people with dementia.

### Aims and Hypotheses

This study aimed to investigate the effect of PF on multiple cognitive outcomes, using a large battery of tests to provide a robust estimation of global cognitive functioning in individuals with dementia and healthy older adults. The areas investigated were general cognitive ability, recognition and recall memory, verbal fluency, visual memory, prospective memory (PM), and retrospective memory (RM). It was hypothesized that those with high levels of PF would have better cognitive performance, irrespective of their health status. The secondary aim of the research was to investigate the differential impact of current PF levels on specific cognitive measures. It was hypothesized that the effect of PA would differ across distinct cognitive domains.

## Materials and Methods

### Design

A cross-sectional case–control method was used to administer a battery of five standardized cognitive tests to a group of people with dementia and a group of controls aged 65 and older. Current levels of PF were assessed using the Physical Fitness Questionnaire (PFQ). Participants also answered demographic and lifestyle questions (for individuals with dementia, most of this information were provided by their family member or carer accompanying them).

### Participants

A total of 70 participants took part in the study (30 with dementia and 40 healthy controls). Inclusion criteria for all participants were age of 65+; dementia participants had to have received an official medical diagnosis, and healthy participants had to be in good health (e.g., no depression or other psychiatric conditions that could affect cognitive performance). Dementia participants were recruited through advertisement via “Join Dementia Research” website, as well as at Alzheimer Cafes, which is a monthly social gathering for individuals with dementia and their carers organized by the Alzheimer Café UK Charity across various locations in London and Surrey. Healthy participants were recruited through the use of advertisement posters, word of mouth and local church and community groups. UCL Ethics Committee granted ethical approval for the study. Each participant provided written informed consent.

### Tests and Procedure

Participants completed the following tests.

#### The MMSE (33)

An assessment of overall cognitive performance. Scores ranged from 0 to 30 with higher scores indicating better performance.

#### The California Verbal Learning Test [CVLT-9 (34)]

A shorter nine-word version of the CVLT-II (35) was used, designed specifically for people with dementia, which provided five different outcomes of interest: list A learning trials 1–5 (CVLT immediate recall), total errors including intrusions and repetitions during recall (CVLT recall errors), recall of words freely (CVLT free recall), recall of words prompted by semantic categories (CVLT cued recall), correct identification of 9 words in a list containing 24 interruption words (CVLT recognition accuracy). Scores for CVLT immediate recall range from 0 to 36 and scores for CVLT free and cued recall ranged from 0 to 9 with higher scores indicating better performance. From the number of correct hits and false positives, a d-prime score was calculated as an unbiased measure of recognition accuracy, with higher scores indicating better accuracy. A higher score for CVLT recall errors indicated more errors during the recall.

#### The Verbal Fluency Test [VF (36)]

The VF test provided a measure of verbal, language, and semantic ability as well as executive control (37). Participants were asked to name as many animals as possible in a 2-min period. A score of 1 was given for each correct animal recalled. Higher scores indicated better performance.

#### The Clock Drawing Test [CD (38)]

Participants were asked to draw a clock with the time reading 10 past 11, providing a measure of visual memory. Scores ranged from 0 to 6, a score of 0 indicating no resemblance to a clock, and 6 indicating a perfect clock, with all numbers in the correct place and the correct time given. Results were interpreted according to Shulman’s six-point scoring system (39).

#### The Prospective and Retrospective Memory Questionnaire [PRMQ (40)]

The questionnaire contained eight items of PM and eight items of RM in the context of everyday life. Independent scores were calculated for PM and RM, ranging from 8 to 40 for each questionnaire, with higher scores indicating lower prospective or RM.

#### Physical Fitness Questionnaire (41)

Participants also completed the PFQ (41), a 15-item questionnaire specially designed for older adults to assess their risk for dementia. Questions address aerobic conditioning, strength, and balance or stability. A cutoff score of 33 was used to group participants into “high” or “low” PF. Scores ranged from 15 to 75, with lower scores indicating better overall PF.

Tests were administered face to face by the researcher in the participant’s home or at an Alzheimer Café. A carer or family member was present to help provide information about the individuals with dementia (such as a history of head injury, or midlife levels of PA). Once inclusion criteria had been met, and written consent had been given, participants answered some questions about their demographics and lifestyle information. They then completed the 15 questions of the PFQ, after which they were guided by the researcher through a battery of five standardized cognitive tests, in the order as presented earlier. The CVLT-9 covered both short- and long-term memory and were administered separately. The VF, CLOCK DRAWING, and PRMQ were completed after the CVLT short-term memory questions, to introduce a temporary delay before the long-term memory questions. The total duration of testing was approximately 1 h.

The lifestyle and demographics questionnaire included questions related to participants’ age, gender, education, any history of head injuries which resulted in hospitalization, previous levels of exercise during midlife (low, moderate, or vigorous levels of PA), and the date of dementia diagnosis (for the dementia group only).

### Statistical Analyses

The demographic information was used to select covariates among age, gender, education, occupation, head injury, and previous levels of exercise in the analyses. A 2 × 2 between-subject multivariate analysis of covariance (MANCOVA) analyses was used to explore the effect of PF and health status (healthy or dementia) on 10 different test measures assessing cognitive performance. Age and previous levels of exercise were included in the models as covariates, as both revealed univariate associations with the dependent measures (see Table S1 in Supplementary Material). Discriminant analysis was used to assess the observed multivariate interaction in the MANCOVA. All analyses were conducted using SPSS version 24.

## Results

Table 1 presents descriptive statistics for the 70 participants grouped according to their health status (healthy and dementia) and PF level. Healthy controls had significantly higher levels of current PF than the dementia group. Individuals with dementia were 5.4 years older than the healthy group. There were more women with low PF compared to men. Interestingly, high levels of past exercise were associated with high PF but were not related to health status.

**Table 1:** Demographics of sample.

		Health status			PF		
		Healthy	Dementia	χ^2^	Low	High	χ^2^
Status	Healthy	–	–	–	14	26	5.52*
	Dementia	–	–		19	11	
Age	Mean (SD)	77.4 (8.2)	82.8 (6.7)	2.96**,^b^	83.2 (7.6)	76.7 (7.1)	3.71***,^b^
Gender	Female	26	16	0.97	24	18	4.21*
	Male	14	14		9	19	
Education^a^	Low	27	14	3.07^+^	20	21	0.11
	High	13	16		13	16	
Employment	Manual	26	21	0.19	20	27	1.21
	Non-manual	14	9		13	10	
Head injury	Yes	3	5	1.42	4	4	0.03
	No	37	25		29	33	
Past exercise	Low/moderate	15	16	1.74	19	12	4.47*
	High	25	14		14	25	
Dementia type	Alzheimer’s	–	19	–			
	Vascular	–	4				
	Frontal lobe	–	2				
	Lewy bodies	–	1				
	Mixed	–	4				

*^a^Low education = Junior school, Secondary school, College; High education = University, Advanced degree*.

*^b^t-Test*.

***p < 0.001, **p < 0.01, *p < 0.05, marginally significant ^+^p < 0.10.

*PF, physical fitness*.

Table 2 presents the means and SDs of the test scores separated by PF level and health status.

**Table 2:** Descriptive and inferential statistics for the univariate analyses.

	Healthy		Dementia		Status^a^	PF^a^	Interaction
			
Test	Low PF	High PF	Low PF	High PF^b^	*F*(1, 64)	*F*(1, 64)	*F*(1, 64)
MMSE	28.4 (2.1)	29.8 (0.4)	19.0 (8.0)	26.5 (4.4)**	26.10**	9.77*	6.56
CD	5.7 (0.6)	5.9 (0.3)	2.8 (1.8)	5.2 (1.3)**	34.03**	12.06**	15.97**
VF	27.6 (9.4)	33.8 (9.3)	11.1 (8.1)	20.4 (9.2)	33.97**	3.85	1.00
Imm	26.5 (6.1)	26.4 (4.5)	11.1 (7.5)	17.8 (6.7)	49.79**	1.54	5.72
Free	6.5 (2.2)	6.6 (2.0)	1.2 (1.8)	2.2 (2.8)	69.57**	0.46	2.16
Cued	7.3 (1.5)	7.4 (1.5)	1.7 (2.0)	3.4 (2.3)	98.89**	0.15	5.17
Errors	2.6 (3.2)	2.6 (3.1)	8.2 (9.1)	10.7 (8.4)	15.15**	1.27	0.52
Recog	3.1 (0.8)	3.0 (0.6)	1.1 (0.9)	1.7 (1.0)	52.80**	0.39	6.96**
RM	16.1 (5.2)	15.9 (3.8)	30.2 (8.3)	25.9 (4.6)	64.40**	2.29	1.55
PM	17.7 (6.3)	17.5 (4.5)	32.1 (8.0)	26.9 (4.6)	56.90**	2.93	2.17

*MMSE, Mini-Mental State Examination; CD, clock drawing test; VF, verbal fluency; Imm, immediate recall; Free, free recall memory; Cued, cued recall memory; Errors, recall errors; Recog, recognition memory (*d*-prime score); RM, retrospective memory; PM, prospective memory*.

*^a^Inferential statistics are based on univariate analyses including the same covariates as the main multivariate analysis of covariance. In order to correct for family-wise error, the significance level is decreased to 0.05/10 = 0.005*.

*^b^When the interaction in the final column is significant, the result of a simple effect analysis is indicated in this column, showing the effect of physical fitness in the dementia group*.

**p < 0.001, *p < 0.005.

A MANCOVA was conducted to assess the effects of PF and health status on the dependent measures, including as covariates age and previous levels of exercise, as these were the only demographic variables that showed at least one significant univariate test result (see Table S1 in Supplementary Material). The pattern of results remained the same when these covariates were excluded from the analysis.

There were significant multivariate effects of health status [Pillai’s Trace^1^ = 0.68, *F*(10, 55) = 11.58, *p* < 0.001, observed power = 1.00] and level of PF [Pillai’s Trace = 0.31, *F*(10, 55) = 2.47, *p* < 0.05, observed power = 0.91], and an interaction between these factors [Pillai’s Trace = 0.28, *F*(10, 55) = 2.13, *p* < 0.05, observed power = 0.85]. To investigate these effects, discriminant analysis (method = enter) was used in which the combined set of 10 measures predicted the health status by the level of PF.

The overall test was significant (Wilks λ = 0.15, χ^2^ = 119.42, df = 30, canonical correlation = 0.87, *R*^2^ = 0.75, *p* < 0.001). Reclassification of cases based on the canonical variables was successful: 71.4% of the cases were correctly reclassified into their original categories, but 54.3% of the cross-validated cases were correctly classified. The misclassifications were predominantly due to dementia cases with high PF being misclassified as healthy (45% compared to 16% for low PF dementia cases), whereas the reverse misclassification was less likely (7% for low PF healthy and 4% high PF healthy).

**Figure 1:**
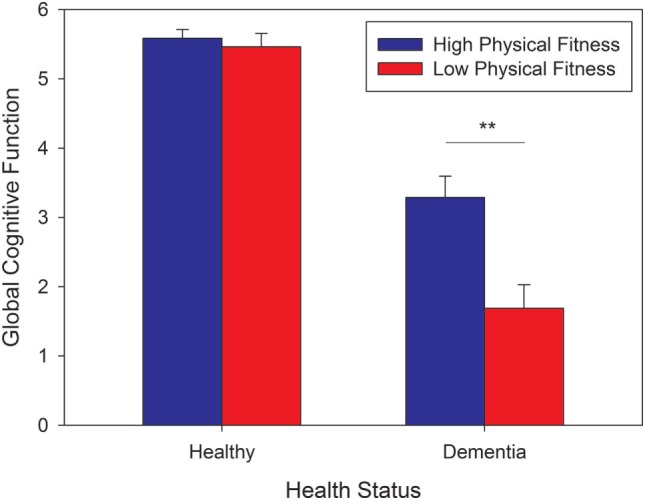
Global cognitive function score as a function of health status and level of physical fitness. The scores were estimated using the first discriminant function, which accounts for 83% of the variance. All scores were referenced to the minimal value of -4.14. Error bars represent standard errors from the mean (SEM).

The analysis extracted two functions (eigenvalues: 3.045 and 0.455), accounting for 95.4% of the variance in cognitive performance, with the first function accounting for 83%. This function was labeled “Global cognition,” as for all dependent measures, better scores increased the value from the discriminant function. Figure 1 shows the resulting values from function 1 against the reference of the minimal value (=−4.14) in the sample. The interaction observed from the MANCOVA reflects better global cognition for dementia cases who are physically fit compared to those who are not, combined with a lack of such an effect for the healthy controls. Using fitted values for each individual, a two-way ANOVA crossing health status and PF revealed main effects of status [*F*(1, 66) = 145.42, *p* < 0.001, partial η^2^ =0.69, observed power = 1.00] and PF [*F*(1, 66) = 11.69, *p* = 0.001, partial η^2^ = 0.15, observed power = 0.92] and an interaction [*F*(1, 66) = 8.58, *p* < 0.01, partial η^2^ = 0.12, observed power = 0.82], due to a significant simple effect of PF for dementia cases [*t*(28) = 3.18, *p* < 0.01], but not for healthy controls (*p* > 0.90). These results remained significant when including age, past exercise, and time since diagnosis (for the dementia group) as covariates.

Whereas the above two-way ANOVA takes into account all dependent measures in a linear-weighted manner, univariate analyses on each measure (corrected for multiple testing) lack the necessary univariate power. The pattern of results differed across dependent measures (see Table 2). All dependent measures revealed better performance for healthy individuals compared to dementia patients (all *p* < 0.001). Only MMSE and clock drawing, exhibited better performance for high compared to low PF. There were interaction effects just for clock drawing, with larger effects of PF for those with dementia in contrast to the control group when taking into account age, past exercise, and years since diagnosis as covariates.

## Discussion

This study explored the influence of PF on cognitive functioning in participants with dementia and healthy controls. As expected, those with dementia showed lower levels of cognitive functioning than healthy group. Of interest are the findings that current PF levels were found to have a significant overall effect on cognition specifically on MMSE and clock drawing.

The association between PF and MMSE is an interesting finding, given that previous research into the effects of PA on MMSE in people with dementia and MCI has provided inconsistent results. For example, whereas some RCTs (42–45) show positive effects of PA on MMSE, Venturelli et al. (46) did not find a strong advantage for their walking intervention. However, the decline in MMSE scores was lower in the intervention group compared to the control group, suggesting a relative attenuation of decline with exercise, although the interaction did not reach significance after correcting for multiple testing.

Performance on the clock drawing task was significantly affected by PF levels in dementia participants but not in control participants. The abilities required for clock drawing depends on executive functions, such as visuoconstructive abilities, spatial function, planning, abstract thinking, and attention (47). This research supports the hypothesis proposed by Kramer et al. (24) that executive control processes showed the most considerable benefit of improved fitness [see also Ref. (23, 25, 48)].

Only clock drawing revealed a reliable interaction between health status and PF, highlighting different sensitivities of the tests to the combination of health status and PF level. The reason for this may be related to the requirement of visual processing which has been shown to be particularly sensitive to dementia-related cognitive decline [e.g., Ref. (49)].

With regard to the demographic information, although high levels of past exercise were associated with high PF, it was not related to health status. This suggests that high levels of PA may not in and of itself reduce the incidence of dementia, but when dementia does occur a history of high levels of physical exercise may limit the amount (or perhaps the rate) of cognitive deterioration. With a cross-sectional design, this study is unable to address this pattern of decline in more detail but provides a direction for longitudinal analyses.

### Potential Biological Mechanisms

Experimental and cohort studies demonstrate that long-term regular PA alters brain structure and cognitive function (50–52).

The differential profile of positive effects on cognitive functioning suggests that the impact of PA is more complex than the argument that PA positively affects cognitive function by enhancing general brain vitality (15). A possible explanation for this could be that PA stimulates blood circulation in frontostriatal circuits, improving executive function in older adults with dementia. Some support for this comes from the finding that PA is associated with angiogenesis and enhanced blood flow in brain regions that are involved in executive functioning (53).

Evidence from animal studies suggests that aerobic exercise increases the levels of growth factors (e.g., BDNF), capillary blood supply to the prefrontal cortex, the growth of new neurons, and as a result enhanced cognitive performance (54, 55). In humans, Colcombe and Kramer (23) reported that 6 months aerobic walking significantly increased activity in the prefrontal cortical areas and enhanced executive function task performance. Furthermore, in a recent prospective study, Tan et al. (56) observed a positive linear association between (self-reported) PA and total brain and hippocampal volumes together with a reduced risk of dementia.

Functional imaging studies that focus on PF found greater hippocampal volumes and denser striatal structures that predicted performance in executive tasks (57, 58). Although this work investigated children, it demonstrates that PF alone is associated with structural brain changes. Manckoundia and colleagues (59) showed that the spatiotemporal dynamics of sit-to-stand and back-to-sit, which are movements involved in the 30-s chair stand test differ between healthy adults and those with dementia. Auyeung et al. (60) demonstrated that the association between PF and cognitive functioning was independent of muscle strength. These findings are interpreted as the neural integrity influences both tests of PF (including strength and balance) and cognitive functioning.

### Strengths and Limitations

In this cross-sectional study, we investigated the associations between PF and cognitive performance. The use of self-report measures of PF represents a potential limitation of this study since we did not have other objective measures available. However, we placed considerable effort in accurately estimating current levels of PF and past levels of PA of the participants with dementia through arranging interviews with family members who knew the individual for a more extended period of time. Furthermore, there is evidence from several studies suggesting that the self-report measures of physical ability tend to be highly correlated with objective test scores (61, 62). Finally, in a recent systematic review, Stephen et al. (63) observed that all 24 prospective and intervention studies they considered used self-report with most (18 out of 24) studies showing a reduced risk of Alzheimer’s disease. Also, the PFQ asks about balance, strength, and aerobic, which bare similarity to ADL, which Forbes et al. (18) showed to be positively associated with objective measures of PA. Future studies could expand the range of metrics used to assess PF, including tests such as the timed up and go test (64) and the 30-s chair stand test (65), to evaluate its link with cognition in healthy people and those with dementia.

This research used an extensive battery of memory tests, each of which has been widely used and validated in dementia research. This is highly useful for providing a robust assessment of cognition and memory, despite being costly in time for the participants and researchers. By using a battery of multiple cognitive tests, a latent construct of global cognitive functioning could be extracted that weights each test accordingly to maximize its classification accuracy. This approach takes into account the differential sensitivity of tests to global cognitive ability. Using a much-extended battery of tests that covers a wider range of cognitive domains would be desirable to address latent constructs of each domain separately.

## Conclusion

Physical fitness is associated with higher global cognition in individuals with dementia, independent of their levels of past exercise and illness duration. This research finds significant positive effects of PF on some, but not all cognitive tests in individuals with dementia. Results from this study support the WHO recommendation for increased PF for older adults in general and those with dementia in particular. Future research should focus on how this can be made accessible to older adults who may have mobility issues, physical disabilities, and cognitive impairment.

## Ethics Statement

This study was carried out in accordance with the recommendations of the UCL Research Ethics Committee with written informed consent from all subjects. All subjects gave written informed consent in accordance with the Declaration of Helsinki. The protocol was approved by the UCL Research Ethics Committee (Project ID Number: 6439/003).

## Author Contributions

DC and AH designed the study and collected the data. DC, AH, and ED analyzed the data and wrote the paper.

## Conflict of Interest Statement

The authors declare that the research was conducted in the absence of any commercial or financial relationships that could be construed as a potential conflict of interest. The reviewer LV and handling editor declared their shared affiliation.

## Footnotes

^1^Pillai’s trace values are reported as the Box’s M test was significant [M = 513.12, F(165, 4,960) = 2.08, p < 0.001].
