# Supplementary material for: Increased Physical Fitness Is Associated with Higher Executive Functioning in People with Dementia
Source: Front Public Health. 2017 Dec 21;5:346. doi: 10.3389/fpubh.2017.00346 (PMC5742628; doi:10.3389/fpubh.2017.00346)
Supplement: Supplementary file 1 [file Table_1.PDF]

Table S1. F-values for all the univariate analyses for the six demographic variables.

Test[a]	Age[b]	Gender	Education	Occupation	Head injury	Past exercise
MMSE	-.26*	0.25	0.15	0.68	0.07	5.74*
CD	-.32**	0.40	0.25	1.89	0.02	6.65*
VF	-.45***	0.24	0.22	0.29	0.00	7.62**
Imm	-.35**	0.80	0.94	1.26	0.02	4.06*
Free	-.53***	0.68	1.01	0.14	0.06	2.43
Cued	-.45***	0.70	1.19	0.21	0.19	5.35*
Errors	.20+	0.05	0.03	0.04	0.57	0.90
Recog	-.51***	0.03	0.16	0.07	0.00	4.79*
RM	.22+	0.30	0.25	0.05	0.55	2.92+
PM	.22+	0.01	0.01	0.16	0.76	2.53

Note: degrees of freedom (1,68). [a] MMSE = Mini-Mental State Examination, CD = Clock drawing test, VF = Verbal Fluency, Imm = Immediate Recall, Free = Free recall memory, Cued = Cued Recall memory, Errors = Recall Errors, Recog = Recognition memory (d-prime score), RM = Retrospective memory, PM = prospective memory. [b] Pearson correlation coefficients. *** $p < .001$, ** $p < .01$, * $p < .05$, + $p < .10$.
